# Stepping down

**DOI:** 10.7554/eLife.54265

**Published:** 2020-01-14

**Authors:** Eve Marder

**Affiliations:** 1 Volen CenterBrandeis UniversityWalthamUnited States; 2 Biology DepartmentBrandeis UniversityWalthamUnited States

**Keywords:** living science, scientific publishing, peer review

## Abstract

As Eve Marder stands down as a Deputy Editor of *eLife*, she reflects on the need for journals to change and respond to their environment

*eLife* has been a major focus of my professional life since 2012, when I was recruited to become one of the first cohort of Senior Editors. As I recall, there were 15 of us – all relatively well-known in our respective fields, and many of us past the age of retirement in some countries. When Randy Schekman asked us to join him in the launch of this ambitious new enterprise, he was hoping that our experience would bring credibility and wisdom to the venture.

I have greatly enjoyed my time at *eLife*. I felt, and continue to feel, that *eLife* is aspiring to be on the side of the angels, to stay committed to excellence, and to honor our individual and collective searches for truth. I have met a large number of interesting people through *eLife*, and have been fascinated by editorial consultation sessions in which referees have argued about the evidence in the manuscripts they have just reviewed. I have been involved in publishing and rejecting many papers. You might be interested to know that we have rejected papers from members of our editorial board, and that while *eLife* has published a number of papers from my laboratory, it has also rejected others – and of course I think some of those rejections were unreasonable. I also like to think that *eLife* has created a community of peers.

Many of the papers *eLife *has published have been important in their fields, but undoubtedly some have been banal. Hopefully, we have published relatively few that are misleading or outright wrong. But, that too is unavoidable, because whenever scientists are attempting to work at the frontiers of what is known, there will be the honest mistakes that occur because we just don’t yet know enough.

For me, one of the strengths of the *eLife* editorial process is that as we grew, we added new editors and new expertise. We have recruited many editors who are considerably younger (and possibly more vibrant) than our original cohort. Some of the original editors have also moved on for all kinds of reasons – to take on new challenges and responsibilities, because of family or personal reasons, or because they didn’t find the role satisfying or engaging – and some have even returned after leaving.

I love the fact that the *eLife* editorial board is in a constant state of flux, and as we have appointed younger and more diverse editors, the board has come to resemble more closely our authors and the scientific community at large. Moreover, as new methods have changed the way that science is both practiced and published, the journal has benefitted from the input of those who have been involved in many of these developments. At the same time, many of our editors have stayed for long enough to create a backbone of stability and transfer the *eLife* ethos to newcomers. The continued presence of more senior scientists on the board also provides the perspective of years of experience and helps us to see the longer view.

**Figure 1:**
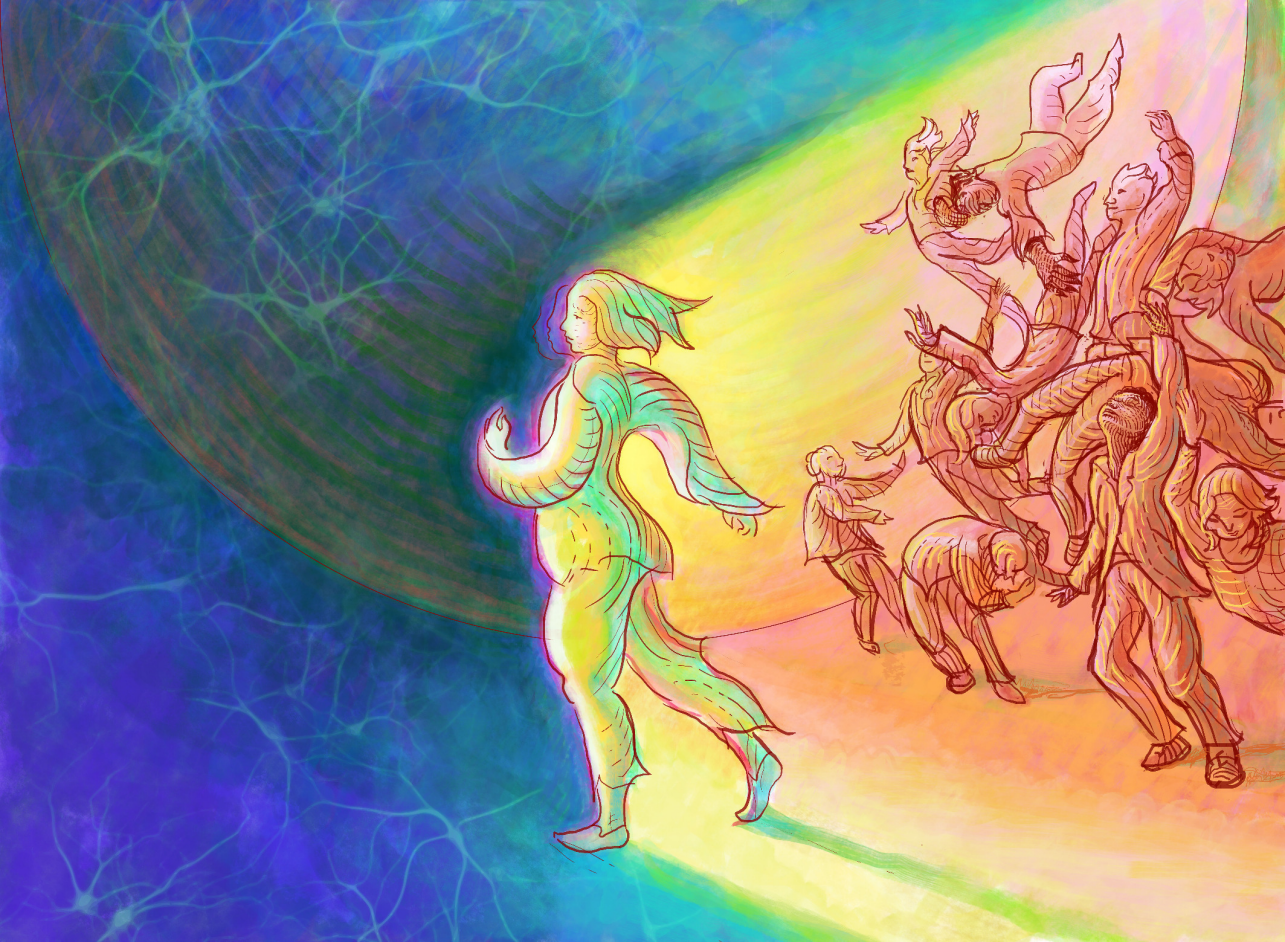
"I see *eLife* as similar to the nervous system, seeking to find and maintain a balance between the need for stability and the need to change."

Illustration: Ben Marder.

I see *eLife* as similar to the nervous system in many ways, seeking to find and maintain a balance between the need for stability and the need to change. We know that human neurons live for many, many years, while all the ion channels and receptors that give them their distinct electrical properties turn-over on timescales that are measured in hours, days or weeks. Moreover, the processes by which neurons regulate the renewal of ion channels and receptors are fundamental and only partially understood. We also know that learning requires changes in various features of neurons and the circuits in which they operate, but that this flexibility must be managed so that it does not destroy the core features and properties of those circuits. Thus, to be successful in the world an animal must be flexible, without compromising its essential operations. So too, for *eLife*.

Just as climate change is creating new and unanticipated challenges for everyone, changes in scientific publishing are major challenges for the scientific community. My fervent wish is that *eLife* will continue to evolve gracefully to meet the future needs of the scientific community. *eLife* will need to take some risks to move forward, and in so doing may discover deleterious unintended consequences. Nonetheless, I am confident that the collective wisdom of our community of editors, reviewers and authors will allow *eLife* to continue to be innovative while continuing to support the publication of outstanding science.

